# Analysis of risk factors for cervical lymph node metastasis of papillary thyroid microcarcinoma: a study of 268 patients

**DOI:** 10.1186/s12902-019-0450-8

**Published:** 2019-11-15

**Authors:** Jian-hua Gu, Yan-na Zhao, Rong-li Xie, Wen-juan Xu, Da-li You, Zhi-feng Zhao, Fei Wang, Jian Fei

**Affiliations:** 1 Department of General Surgery, Shanghai Ruijin Rehabilitation Hospital, Shanghai, China; 2 Department of Ultrasound, Shanghai Ruijin Rehabilitation Hospital, Shanghai, China; 3 Department of Surgery, Luwan Branch, Ruijin Hospital affiliated to Shanghai Jiao Tong University School of Medicine, Shanghai, China; 4 Department of Critical Care Medicine, Jiading District Central Hospital Affiliated Shanghai University of Medicine & Health Sciences, Shanghai, China; 5 Department of General Surgery, Ruijin Hospital, Shanghai Jiao Tong University School of Medicine, Shanghai, China

**Keywords:** Papillary thyroid microcarcinoma, Cervical lymph node metastasis, Cervical lymph node dissection

## Abstract

**Background:**

To investigate the risk factors of cervical lymph node (LN) metastasis in papillary thyroid microcarcinoma (PTMC) patients.

**Methods:**

We retrospectively analyzed the clinicopathologic data of all patients who received standard lobectomy for PTMC at our institution between October 2017 and January 2019. Central LNs were dissected in all patients. Lateral LNs were dissected if metastasis to the lateral LNs was suggested based on pre-op fine-needle aspiration biopsy. The relationship between variables available prior to surgery and cervical LN metastasis was examined using multivariate regression.

**Results:**

Post-op pathologic examination revealed cervical LN metastasis in 79 (29.5%) patients. Seventy subjects had metastasis only to central LNs, and 4 (1.5%) patients had metastasis only to lateral LNs. Five patients had metastasis to both central and lateral LNs. In comparison to patients without cervical LN metastasis, those with LN metastasis were significantly younger (40.63 ± 13.07 vs. 44.52 ± 12.23 years; *P* = 0.021) and had significantly larger tumor diameter on pathology (6.7 ± 2.2 vs. 5.9 ± 2.4 mm; *P* = 0.010). Multivariate regression analysis identified the following independent risks for cervical LN metastasis: male sex (OR 2.362, 95%CI 1.261~4.425; *P* = 0.007), age (OR 0.977, 95%CI 0.956~0.999; *P* = 0.042) and ultrasound tumor diameter at > 5 mm (OR 3.172, 95%CI 1.389~7.240; *P* = 0.006).

**Conclusion:**

Cervical LN metastasis occurs in a non-insignificant proportion of PTMC patients. Independent risks included male sex, younger age and larger tumor diameter on ultrasound.

## Background

According to the World Health Organization (WHO) report, the number of new cases of thyroid cancer in China accounted for 15.6% of that worldwide, and the number of deaths accounted for 13.8% [1]. Papillary thyroid carcinoma (PTC) is the most common pathological type of thyroid malignancy and accounts for about 85 to 90% of all cases of thyroid malignancies. PTC with a maximum tumor diameter of 10 mm or less is defined as papillary thyroid microcarcinoma (PTMC) [2]. Several studies have indicated that PTMC has a low rate of recurrence and metastasis [3] as well as an extremely high 10-year survival rate [4]. In recent years, improvements in diagnostic methods such as imaging and ultrasound-guided fine needle aspiration biopsy have led to a significant increase in the diagnosis rate of PTMC [5].

The most common site of PTMC metastasis is cervical lymph nodes, especially the central lymph nodes (known as level 6) [6, 7]. Surgery remains the main therapeutic modality for PTMC: it is a consensus that patients with cervical LN metastasis be managed by LN dissection; however, the necessity of LN dissection in patients with clinically negative lymph nodes (cN0) has been debated [8]. Active surveillance data from Japan suggested that even those at risk for disease progression such as young age and pregnancy should be actively monitored instead of surgery [2]. On the other hand, cervical LN metastasis increases the risk of loco-regional recurrence of PTC [9]. As a result, it is important to identify predictors of cervical LN metastasis.

In the current retrospective analysis, we examined the potential correlation between pre-operative variables with cervical LN metastasis in 268 PTMC patients.

## Methods

The current study (including access to raw data) was approved by the Ethics Committee of Shanghai Ruijin Rehabilitation Hospital (Committee Chairman: Ms. Yuezhen Dai) and performed in accordance with the Declaration of Helsinki and Good Clinical Practice guidelines. Patient consent was not required because of the retrospective nature of the study. Anonymized patient data (in the Chinese language) are available upon request.

### Patients

This retrospective study analyzed the clinical data of pathologically proven PTMC patients who were initially treated at Shanghai Ruijin Rehabilitation Hospital, Shanghai, China, between October 2017 and January 2019. Patients were included if 1) they received initial diagnosis and treatment for thyroid nodules; 2) they were diagnosed with PTMC by preoperative fine-needle biopsy and underwent conventional lobectomy of thyroid carcinoma; 3) they had pathologically proven solitary PTMC; 4) the maximum tumor diameter on pathology was ≤10 mm.

### Patient evaluation

The following data were retrieved from the hospital electronic record systems: name, sex and age and clinicopathologic and surgical data including 1) time to surgery from initial presentation; 2) surgical procedure received; 3) pathological type, maximum diameter and location (classified as inferior, middle, superior, or isthmus within the thyroid gland) of the tumor; 4) histological results of involved central and/or lateral lymph nodes; 5) concurrent Hashimoto’s thyroiditis; 6) routine laboratory results such as blood chemistries, thyroid function, parathyroid hormone and blood calcium before and following operation.

All patients underwent preoperative physical examination, high-quality thyroid ultrasonography (US), and US-guided fine-needle aspiration biopsy of suspected thyroid nodule or lymph nodes. Solitary PTMC was considered if the tumor showed no pathological evidence of multifocality within the thyroid gland. The maximum diameter and location of the primary tumor within the thyroid were determined by pathological examination.

### Surgery

All patients underwent standard lobectomy. Central LN dissection was conducted in all subjects. In patients suspected of metastasis to lateral LNs based ultrasound examination prior to surgery, lateral LNs were also dissected. The excised tissue was then sent for pathological examination. Patients received routine rehydration as well as symptomatic treatment postoperatively and were closely observed for incision hemorrhage, hoarseness, or numbness.

### Statistical analysis

The data was analyzed with SPSS 20.0 software (SPSS Inc., Chicago, IL, USA). Student’s *t****-***test and χ^2^ test were used to examine potential differences between subjects with vs. without cervical LN metastasis. Multivariate logistic regression was used to evaluate the correlation between pre-operative variables and cervical LN metastasis. *P* < 0.05 was considered statistically significant.

## Results

### Patient demographic and baseline characteristics

**Fig. 1:**
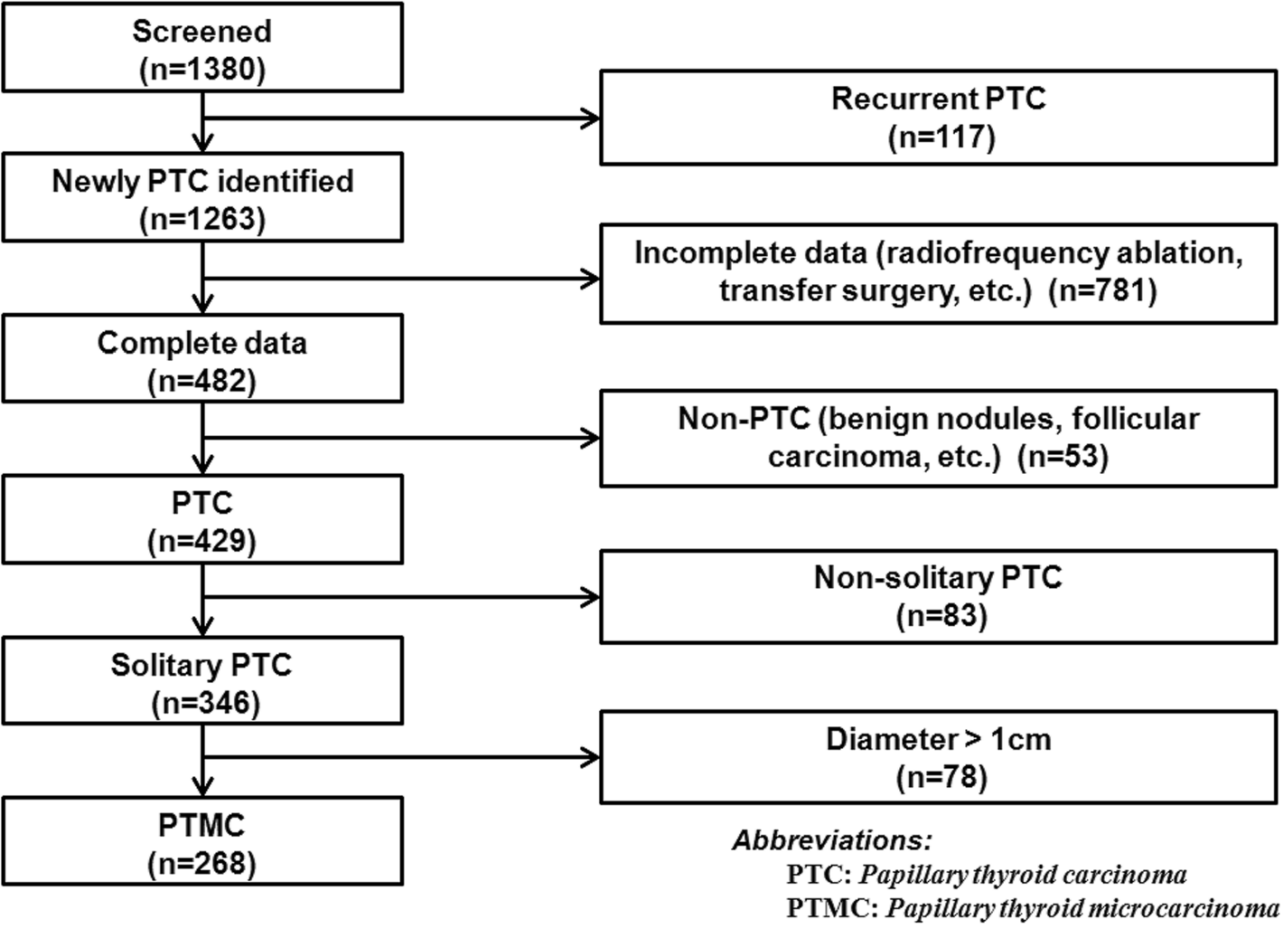
The study flowchart

The study flowchart is shown in Fig. 1. A total of 429 patients with a diagnosis of primary PTC with complete data were screened. Eighty-three patients with non-solitary lesion were excluded and 78 patients were excluded because the maximum tumor diameter on pathology was > 10 mm. The final analysis included 268 patients (208 women; mean age 43.3 ± 12.6 years, range 15–72). Demographic and baseline variables are shown in Table 1.

**Table 1:** Patient demographic and baseline characteristics

Variables	All (*n* = 268)
Female sex, n(%)	208(77.6)
Age, years	
Mean ± SD	43.3 ± 12.6
Range	15, 72
Maximum diameter on pathology, mm	
Mean ± SD	6.1 ± 2.4
Range	1, 10
> 5, n(%)	148 (55.2)
Maximum diameter on ultrasound, mm	
Mean ± SD	9.8 ± 6.8
Range	2, 49
≤ 5	53 (19.8)
> 5	215 (80.2)
Location, n(%)	
Superior	69 (25.7)
Middle	99 (36.9)
Inferior	77 (28.7)
Isthmus	15 (5.6)
Missing	8 (3.0)
Hashimoto’s thyroiditis	
Yes	79 (29.5)
Capsular invasion, n(%)	
Yes	52 (19.4)
Extrathyroidal extension, n(%)	
Yes	49 (18.3)
Cervical LN metastasis, n(%)	79 (29.5)
Only central	70 (26.1)
Only lateral	4 (1.5)
Both central and lateral	5 (1.9)

### Lymph node metastasis

Seventy-nine (29.5%) patients had cervical LN metastasis: 70 subjects had metastasis only to central LNs, and 4 (1.5%) patients had metastasis only to lateral LNs. Five patients had metastasis to both central and lateral LNs. Compared to patients without LN metastasis, those with cervical LN metastasis were significantly younger (40.63 ± 13.07 vs 44.52 ± 12.23 years; *P* = 0.021) and had significantly larger tumor diameter on pathology (6.7 ± 2.2 vs. 5.9 ± 2.4 mm; *P* = 0.010) (Table 2). The two groups were comparable in other demographic and baseline variables.

**Table 2:** Demographic and baseline characteristics in subjects with vs. without cervical LN metastasis

Variables	Yes	No		*P*
N.	79	189		
Female sex, n(%)	53 (67.1)	155 (82.0)		0.008
Age, years				
Mean ± SD	40.63 ± 13.07	44.52 ± 12.23		0.021
Range	15,67	22,72		
Maximum diameter on pathology, mm				
Mean ± SD	6.7 ± 2.2	5.9 ± 2.4		0.010
Range	2, 10	1, 10		
≤ 5	29 (36.7)	91 (48.1)		0.086
> 5	50 (63.3)	98 (51.9)		
Maximum diameter on ultrasound, mm				
Median (interquartile range)	9.1 (6.9~11.2)	7.8 (5.4~10.6)		0.015
Range	3, 45.8	2, 49		
≤ 5	8 (10.1)	45 (23.8)		0.010
> 5	71 (89.9)	144 (76.2)		
Location, n(%)				0.957
Superior	21 (27.6)	48 (26.1)		
Middle	29 (38.2)	70 (38.0)		
Inferior	21 (27.6)	56 (30.4)		
Isthmus	5 (6.6)	10 (5.4)		
Missing	8 (3.0)			
Hashimoto’s thyroiditis			0.248	0.618
Yes	19 (20.3)	51 (27.0)		
Capsular invasion			0.321	0.571
Yes	17 (21.5)	35 (18.5)		
Extrathyroidal extension			0.785	0.376
Yes	17 (21.5)	32 (16.9)		

### Risks of cervical LN metastasis

A univariate analysis showed that cervical LN metastasis is associated with the male sex (*P* = 0.008), younger age (*P* = 0.021) and larger tumor diameter on ultrasound (*P* = 0.010). The multivariate analysis confirmed the following risks: male sex (OR 2.362, 95%CI 1.261~4.425; *P* = 0.007), younger age (OR 0.977, 95%CI 0.956~0.999; *P* = 0.042) and ultrasound tumor diameter at > 5 mm (OR 3.172, 95%CI 1.389~7.240; *P* = 0.006) (Table 3).

**Table 3:** Multivariate analysis of risk factors for cervical LN metastasis

	OR	95%CI	*P*
Male sex	2.362	1.261~4.425	0.007
Age	0.977	0.956~0.999	0.042
Maximum diameter on ultrasound, > 5 mm	3.172	1.389~7.240	0.006

The analysis included a total of 268 subjects: 79 with cervical LN metastasis

## Discussion

In the current study, approximately 30% PTMC patients had cervical LN metastasis. Independent risk factors included the male sex, younger age and tumor size on ultrasound at > 5 mm. Given the fact that cervical LN metastasis increases the risk of loco-regional recurrence of PTC [9], our findings suggest that this non-insignificant proportion of at risk PTMC patients should be actively monitored. Based on these findings, we believe that LN dissection is necessary in young men with PTMC with tumor diameter at > 5 mm. Skip metastasis was identified in 4 out of the 268 cases. We therefore did not attempt an analysis to examine the risk of skip metastasis.

In recent years, there has been a noticeable increase in the incidence of thyroid malignancies in China and globally, in which PTMC accounts for a large proportion of all thyroid cancer cases [10]. The rate of cervical LN metastasis in PTMC patients has been reportedly from 12 to 64% [7]; the rate of cervical LN metastasis in our study (29.5%) falls within this range. Cervical LN metastasis is associated with tumor stage, invasiveness and recurrence rate, and serves as one of the essential prognostic predictors of PTMC [11, 12]. Although thyroid malignancies are now diagnosed at an early stage owing to recent advances in imaging technologies, image reading can be influenced by multiple factors such as tumor characteristics and physicians’ skills.

In current consensus, LN dissection should be performed in PTMC patients with pathologically confirmed cervical LN metastasis. Whether cervical LNs should be routinely dissected in cN0 PTMC patients remains controversial. A previous study suggested that LN dissection had no significant effect on the recurrence and metastatic rate of PTMC [13].

Based on previous literature, independent risk factors of cervical LN metastasis in PTMC patients include age over 45 years [14], male sex [14, 15], larger tumor diameter [16] and capsule invasion [6, 7, 16]. Other clinicopathologic variables associated with cervical LN metastasis may include lesion location [17], multifocality (the number of lesions no less than 2) [18–21], and concurrent Hashimoto’s thyroiditis [15, 22]. Our study focused on solitary PTMC and found that tumor diameter on ultrasound at > 5 mm is an independent risk of cervical LN metastasis in PTMC (OR = 3.172, *P* = 0.006). The current study also revealed an association between cervical LN metastasis with younger age (OR = 0.977, *P* = 0.042) and the male sex (OR = 2.362, *P* = 0.007). We failed to show an association between cervical LN metastasis with tumor location, capsule invasion or Hashimoto’s thyroiditis, possibly due to insufficient sample size.

The availability of gene sequencing data may allow molecular stratification of cancer patients. Lai et al. showed in a meta-analysis that BRAF^V600E^ mutation was associated with extra-thyroid infiltration, lymph node and distant metastasis, and advanced TNM staging. Therefore, determination of *BRAF* mutation status preoperatively may allow preoperative evaluation of capsule invasion and cervical LN metastasis. However, more studies are required to validate of molecular markers such as *BRAF*.

With the development of medical technology, PTMC patients now have more options for treatment. In addition to the more aesthetically pleasing surgical methods such as thyroidectomy with laparoscopy or robot-assisted surgery, radiofrequency ablation therapy has also garnered the interest of clinicians. Based on the findings from the current study, we believe that ablation may be suitable for selected patients (older age, women, tumor diameter at ≤5 mm) with low risk of cervical LN metastasis.

## Conclusion

Cervical LN metastasis is not a rare occurrence in PTMC patients. Independent risks for metastasis include the male sex, younger age and tumor diameter on ultrasound at > 5 mm.

## Data Availability

The data that support the findings of this study are available from the corresponding author upon reasonable request.

## Abbreviations

BRAF: V-raf murine sarcoma viral oncogene homolog B1
LN: Lymph node
PTC: Papillary thyroid carcinoma
PTMC: Papillary thyroid microcarcinoma
US: Ultrasonography
